# Pathogenic Mechanisms Involved in the Hematological Alterations of Arenavirus-induced Hemorrhagic Fevers

**DOI:** 10.3390/v5010340

**Published:** 2013-01-21

**Authors:** Mirta Schattner, Leonardo Rivadeneyra, Roberto G. Pozner, Ricardo M. Gómez

**Affiliations:** 1 Laboratory of Experimental Thrombosis, Institute of Experimental Medicine, CONICET, National Academy of Medicine. Pacheco de Melo 3081 (1425), Buenos Aires, Argentina. E-Mails: mschattner@hematologia.anm.edu.ar; leonardorivadeneyra@yahoo.com.ar (L.R.); rpozner@hematologia.anm.edu.ar (R.G.P.); 2 Laboratory of Animal Virus, Institute of Biotechnology and Molecular Biology, CONICET-UNLP, Calle 49 esq 115 (1900), La Plata, Argentina. E-Mail: rmg@biol.unlp.edu.ar (R.M.G.)

**Keywords:** Hemostasis, endothelium, platelets, megakaryocytes, coagulation, thrombocytopenia, interferon, nitric oxide.

## Abstract

Viral hemorrhagic fevers (VHFs) caused by arenaviruses are acute diseases characterized by fever, headache, general malaise, impaired cellular immunity, eventual neurologic involvement, and hemostatic alterations that may ultimately lead to shock and death. The causes of the bleeding are still poorly understood. However, it is generally accepted that these causes are associated to some degree with impaired hemostasis, endothelial cell dysfunction and low platelet counts or function. In this article, we present the current knowledge about the hematological alterations present in VHF induced by arenaviruses, including new aspects on the underlying pathogenic mechanisms.

## 1. Introduction

The *Arenaviridae *family*, *whose prototype is lymphocytic chorio­meningitis virus (LCMV), contains more than 20 members with diverse geographical distributions [1]. The arenaviruses are essentially rodent-borne viruses. LCMV infects *Mus musculus*, the common mouse, which explains why this virus is the only arenavirus with a worldwide distribution. In contrast, the other arenaviruses infect different types of rodents with circumscribed geographical distribution patterns that relate to the distribution of the associated viruses. In the rodent, arenaviruses usually establish a persistent chronic infection with few symptoms [1]. However, arenaviruses may occasionally be transmitted to humans through material contaminated with an infected rodent’s excreta. Historically, five types of arenavirus have been associated with hemorrhagic fever (HF): Junin virus (JUNV), the etiologic agent of HF in Argentina (AHF); Machupo virus (MACV), the etiologic agent of HF in Bolivia, Guanarito virus (GTOV), the etiologic agent of HF in Venezuela; Sabia virus (SABV), the etiologic agent of HF in Brazil (HFB); and Lassa virus (LASV), the etiologic agent of HF in west Africa (Lassa fever) [1]. More recently, new arenaviruses have been associated with HF, such as Chapare virus in Bolivia [2] or Lujo virus (LUJV) in Southern Africa [3]. In this regard, arenaviruses are etiologic agents of emerging diseases as a result of environmental modifications by humans, through the creation of new ecological environments either for agricultural production or for places to live that favor contact with wild rodents [4].

In addition to other chapters in this volume, many reviews have been published in recent years about arenaviruses and their pathogenesis [1,5,6,7,8,9]. In this chapter, we will review the data regarding the pathogenesis of arenaviral hemorrhagic fevers (AVHF), with a particular emphasis on the very recent data involving new mechanisms involved in hematological alterations. 

## 2. Hemostasis

The hemorrhagic complications of the South American HF are almost identical regardless of the virus responsible for the disease and consist mainly of petechiae, conjunctival hemorrhages, and mucosal and gastrointestinal bleeding with melena that usually start after 5 days of illness [10]. Although the number of reported cases of AHF has dramatically declined after the introduction of the attenuated vaccine Candid #1 [11], still remains as the best HF characterized of the South American cases. Therefore, most of the available data presented here are related to this disease. 

**Figure 1:**
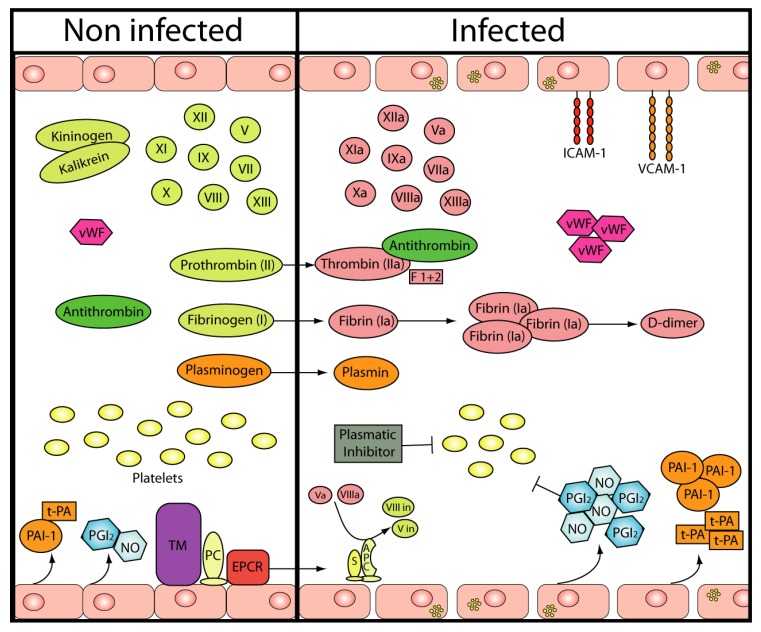
**Hemostasis in AHF.** The picture represents the *in vivo and in vitro* cellular and plasmatic alterations described in the AHF. Endothelial cells are susceptible to Junin virus (JUNV) infection and become activated after virus replication. Cell adhesion molecules (ICAM-1 and VCAM-1) NO, PGI_2_, t-PA and PAI-1 production are enhanced. The level of most of the coagulation factors is decreased, with the exception of Factor V and vWF. However, the Thr/AT complexes and prothrombin fragment 1+2 are augmented, together with high levels of relevant components of the fibrinolytic cascade. Natural inhibitors, such as antithrombin III, protein C and total and free protein S, have been shown to be normal or slightly decreased in AHF patients. Thrombocytopenia is one of the most relevant clinical features in AHF patients and platelet function may probably be inhibited by a plasmatic inhibitor not yet characterized and/or NO and PGI_2_.

Different studies in patients with AHF have shown that regardless of the severity of the clinical form of the disease (mild, moderate or severe), the profiles of the coagulation factors during the course of the disease are similar in all patients, indicating that there is no correlation between the severity of the disease and an impairment of coagulation. In addition to thrombocytopenia, the patients present with several alterations in both the blood coagulation and the fibrinolytic systems, but disseminated intravascular coagulation (DIC) has not been demonstrated. The most consistent alterations of the hemostatic system observed in AHF patients during the early stages include a prolongation of partial thromboplastin with low levels of factors VIII, IX and XI but enhanced activity of factor V. The factor VIII procoagulant activity (F VIII:C) and the F VIII:C antigen are low in the early stages of the disease but increase progressively in the later days. In contrast, the levels of von Willebrand factor (vWF) (VIII related antigen) remain high throughout disease progression and return to normal values during the convalescence period. Thrombin/antithrombin complexes (AT) and prothrombin fragment F1 + 2 levels were also increased in patients at admission, indicating the generation of FXa and thrombin. Neither fibrin monomers nor fibrinogen degradation products were detected, indicating that the hemostatic abnormalities in AHF are not associated with DIC. Moreover, the activation of coagulation in AHF appears to be a limited phenomenon because natural inhibitors, such as antithrombin III, protein C and total and free protein S, have been shown to be normal or slightly decreased in these patients [12,13,14] (Table 1 and Figure 1).

**Table 1:** Hemostatic and vascular alterations in Argentine hemorrhagic fever (AHF).

**Coagulation/Fibrinolysis **		**Platelets**	
Factor VIII	⇩		
Factor IX	⇩	Count (*in vivo* and *in vitro*)	⇩
Factor XI	⇩	Function (*in vivo) *	Not determined
Factor V	⇧		
vWF	⇧		
Thr/AT complexes	⇧	Endothelium	
Prothrombin fragment 1+2	⇧	Viral replication	Yes
FDPs	Not detected	Vascular lesions	No
AT III	**=** or slightly ⇩	Cell adhesión molecules	⇧
Protein C	**=** or slightly ⇩	NO	⇧
Free protein S	**=** or slightly ⇩	PGI_2_	⇧
t-PA	⇧	vWF	⇩
PAI-1	⇧		
D-dimer	⇧		

The fibrinolytic system is also altered in AHF as the tissue plasminogen activator (t-PA) and D-dimer levels are reported to be high, while PAI-1 has been shown to be considerably increased in severe cases. The plasminogen antigen level and functional activity were found to be reduced in the moderate and severe groups. Functional and antigen α2-antiplasmin, α2-macroglobulin and α1-antitrypsin have been shown to be normal or slightly above the normal range [15]. Overall, these data indicate that a low-level but persistent process of blood coagulation and fibrinolysis activation occurs in this viral hemorrhagic disease (Table 1 and Figure 1). 

Lassa fever is classified as a HF, but clinical diagnosis is difficult because obvious bleeding is often absent, even late in the course of the illness. Hemorrhagic manifestations, largely limited to the mucosal surfaces, only occur in 1/3 of the patients and are associated with death [16,17]. There is no data showing evidence of DIC in severe Lassa, as coagulation markers are almost always within the normal range. 

LUJV was identified in 2008 after an outbreak of severe HF in Southern Africa. Although limited data available, it was reported that LUJV-infected patients presented thrombocytopenia and coagulopathy [3]. Interestingly, it was recently demonstrated that after the infection with LUJV, Strain 13/N guinea pigs develop a HF syndrome similar to the disease observed in human patients including pan-leukopenia, thrombocytopenia and profound anemia. Although coagulation studies were not performed, observation of fibrin deposition and hemorrhages in multiple organs together with a marked reduction in platelet counts and tissue damage suggested that DIC was present in LUJV-infected guinea pigs. Moreover, it was suggested that LUJV infection in guinea pigs appears to cause a more severe disease than JUNV or Lassa infection; however, direct comparison studies are required to confirm this hypothesis [18].

## 3. Endothelium

Clinical and experimental data indicate that the vascular endothelium is directly or indirectly involved in the pathogenesis of AVHF (reviewed in [9,19]). Although hemorrhages are not a salient feature of Lassa fever, perturbation of vascular function is likely central to Lassa fever pathology; studies in human patients and non-human primates revealed endothelial cell function failure with an impairment of the regulation of vascular permeability preceding the onset of shock and death [20]. Similar findings were shown in a experimental hamster model infected with the new world arenavirus Pichinde (PICV) [21]. However, no specific vascular lesions were observed in a post-mortem examination of fatal human cases of Lassa fever or in non-human primates experimentally infected with LASV [22,23] or in AHF or experimentally JUNV-infected animals (reviewed in [7]). These discrepancies could be related to the fact that despite an estimated 3,000 fatal cases of LF per year in West Africa, there have been relatively few postmortem histologic or immunohistochemical studies.

The receptors for arenaviruses α-dystroglycan and transferrin receptor 1, and the recently described endothelial calcium-dependent lectin (LSECtin), from the C-type lectin family are highly expressed on vascular endothelial cells (EC) [24,25], and productive infection of LASV and JUNV have been observed *in vitro *in this cell type [26,27]. Moreover, as with other arenaviruses, JUNV and LASV have a non-lytic cell cycle and cause no overt cytopathic effects in cultured vascular EC suggesting that major signs of the associated pathology are mostly attributed to the host response rather than to a direct virus-induced structural damage. [27,28].

A productive infection of EC in culture with JUNV induces the expression of ICAM-1 and, to a lesser extent, VCAM-1 [28]. This up-regulation of the cell adhesion molecules involved in EC activation strictly depended on viral replication, as no effect was observed with a UV-inactivated virus. Because the expression of cell adhesion molecules on the endothelium is a key event in the recruitment of inflammatory leukocytes, it could be possible that the adhesion of activated leukocytes to the endothelium contribute to the increase in vascular permeability in JUNV- and LASSA- infected patients.

*In vitro, *the infection of EC with JUNV resulted in reduced expression and secretion of coagulation factors, such as the prothrombotic vWF. This finding seems to be in contrast to clinical data showing increased vWF in the sera of AHF patients [14]*.* These differences could be explained if serum samples had been collected at successive post-infection times and/or if the source of raised vWF serum levels was not only the EC but also megakaryocyte or platelet population. In addition, the infection of EC with a virulent strain of JUNV, but not a non-virulent isolate, markedly induced the production of the vasoactive mediator nitric oxide (NO) and prostacyclin (PGI_2_) [28], providing a possible link between viral infection and the increased vascular permeability observed in fatal AHF cases. Interestingly, PICV induces microvascular endothelial cell permeability through the production of NO [29], giving further support to the important role of NO in the pathogenesis of the endothelium dysfunction present in AVHF (Table 1 and Figure 1). 

The mechanisms by which LASV affects EC biology, including the putative role of NO, are unknown. A perturbation of the endothelium may include direct effects of the virus involving virus infection and gene expression and/or may occur in an indirect manner by a virus-induced release of host-derived factors that affect endothelial function. In this sense, it has been suggested that a deregulated and ineffective cytokine response, leading to high levels of the virus and pro-inflammatory cytokines in the late stage of the disease, is important in the pathogenesis of hemorrhage and shock in Lassa fever [30]. However, the detection of pro-inflammatory cytokines in the sera of patients with fatal Lassa fever has revealed little evidence for a “cytokine storm” associated with lethal diseases [31]. The molecular mechanisms underlying the cytokine deregulation are not yet elucidated but it is suggested that viral infection leads to disruption of early host defenses and contributes to arenavirus pathogenesis. In this context, it has been recently reported that the nucleoprotein encoded by representative members of both, Old and New World arenaviruses interferes with NF-κB activation, possible contributing to the multiple mechanisms by which arenaviruses counteract the host initial innate defenses and subsequent adaptive immune responses [32]. In this sense it has been shown that in the absence of cell damage, a LASV infection in HUVEC resulted in reduced levels of interleukin (IL)-8, a cytokine synthetized through a NF-κB-dependent pathway [27]. 

Currently, the extent of the infection of the vascular EC by LASV and the consequent effects remain largely unknown. 

## 4. Platelets

Thrombocytopenia, a condition in which the blood has a lower than normal number of platelets, is one of the most consistent findings among human patients and experimental animal models of AVHF; thrombocytopenia is used as a major diagnostic feature in patients with AVHF [33,34]. In Venezuelan HF, for example, most patients showed thrombocytopenia, and although the clinical courses of these patients varied, the gross and histopathological necropsy findings were remarkably similar and generally showed evidence of bleeding [35,36]. In contrast to Lassa fever, the bleeding that occurs with severe thrombocytopenia is more common in Argentine and Bolivian HFs [37,38].

The causes of the thrombocytopenia associated with AVHF remain poorly understood. In this regard, DIC could explain platelet consumption; nevertheless, the occurrence of DIC in AVHF infections is inconclusive, at least for the *arenaviridae* family [34]. Furthermore, the occurrence of thrombocytopenia before the appearance of antibody or complement activation does not support immunologically mediated mechanisms of platelet destruction [39]. Therefore, a high level of splenic sequestration or impaired megakaryo/thrombopoiesis could be the major physiopathogenic mechanisms responsible for the low platelet count. 

Conflicting data were obtained from AHF patients in the 1970s. While Gallardo *et al*. found hypocellularity in bone marrow samples of AHF patients, particularly in the erythroid and megakaryocytic lineages [40], Ponzinibbio *et al.* could not show any megakaryocytic anomalies [41]. However, infected megakaryocytes has been observed in JUNV-infected guinea pigs [42]. 

Recent findings show that JUNV not only replicates in human megakaryocytes and their precursor CD34+ cells but also that viral infection selectively impairs thrombopoiesis by decreasing *in vitro* proplatelet formation and platelet release [43]. The decrease in platelet release was shown to be TfR1-dependent and mimicked by poly(I:C); additionally, type I interferon (IFN I) was implicated as a key paracrine mediator. Although the molecular basis governing the IFN I-mediated reduction of *in vitro* platelet production is still unknown, a low content of NF-E2 (a transcription factor that plays a major role in terminal differentiation of megakaryocytes and platelet release) was found in megakaryocytes treated with IFN I. Moreover, an ultrastructural analysis revealed that in the IFN I-treated megakaryocytes, the distinctive demarcation membrane system was almost absent and lacked organization and platelet territories [43]. Interestingly, a correlation between high levels of circulating IFN α and both virulence and prognosis has been described in clinical [44] and experimental [45] AHF. Overall, these data support an emerging role for IFN I as a pathogenic factor for the thrombocytopenia observed in VHF patients and maybe in other diseases associated with increased bone marrow IFN I levels [43]. 

In addition to a low platelet number, platelet dysfunction might also be a major contributor to the hemorrhagic phenotype observed in AVHF patients. Platelet dysfunction has several potential causes, including circulating fibrin degradation products, activated platelets (exhausted platelets syndrome), or specific inhibitors. In the case of inhibitors, a plasma inhibitor of platelet function was found in 80% of Lassa fever patients with a hemorrhage but in only 16% of those without a hemorrhage and was significantly associated with disease severity [46]. Furthermore, a continuous rise in the inhibitory activity correlated with clinical deterioration, whereas a decline corresponded to clinical improvement. A similar inhibitor of platelet function has been demonstrated in patients with AHF [47]. This inhibitor has *in vitro* effects similar to those observed in patients with Lassa fever; however, it appears to be more thermolabile, and the inhibitory activity was not neutralized by convalescent plasma containing a high titer of protective antibodies against JUNV [48]. Although the presence of a platelet inhibitor could account for the bleeding diathesis, there has been no report describing abnormalities of platelet function in AVHF infected patients, perhaps related to the absence of on-site adequate equipped laboratories of hemostasis (Table 1 and Figure 1).

In 2008, two major advances using experimental mouse models contributed to the current knowledge regarding the role of platelets in AVHF. First, it was demonstrated that mice rendered thrombocytopenic only suffered localized hemorrhages at the sites undergoing non-infectious inflammatory processes and that low numbers of circulating platelets were able to prevent these inflammation-induced hemorrhages [49]. Second, Iannacone *et al.* reported that platelet-depleted mice infected with LCMV (Armstrong strain) developed a syndrome characterized by mucocutaneous bleeding, vascular leakage, anemia, uncontrolled viral replication, suboptimal immune responses, and subsequent death. Remarkably, a lethal hemorrhage was less associated with thrombocytopenia and instead was more closely associated with the platelet dysfunction mediated by high IFN I levels [50]. Interestingly, as neither Interferon-α nor β inhibited platelet responses *in-vitro*, the authors suggested that IFN I could directly affect megakaryocytes rather than platelets. The recent description of the functional IFN I receptor in human megakaryocytes further strengthens this hypothesis [51]. 

Another major issue of the Iannacone *et al.* study was the observation that in addition to having life-threatening hemorrhagic anemia, the platelet-depleted mice failed to mount an efficient cytotoxic T lymphocyte (CTL) response and were unable to clear the LCMV. Transfusion of functional platelets into these animals reduced the hemorrhage, prevented death and restored the CTL-induced viral clearance in a manner partially dependent on the CD40 ligand (CD40L). These results indicate that, upon activation, the platelets expressing integrin β3 and CD40L are required to protect the host against the induction of an IFN I-dependent lethal hemorrhagic diathesis and for clearing the LCMV infection through CTLs. In the same line of evidence, Loria *et al.* recently showed that mice profoundly depleted of platelets (>95% depletion) and infected with the Armstrong LCMV strain developed hemorrhagic spots in several organs along with high viral titers, generalized splenic necrosis, and increased mortality. Interestingly, the authors also found that the presence of the remaining 15% of the platelets was sufficient to prevent vascular damage but not viral replication, necrotic destruction of innate and adaptive immune splenocytes, or CTL exhaustion [52]. These observations not only confirm the novel notion that platelets are necessary to protect vascular integrity and are critical mediators of viral clearance but also underscore an underappreciated relationship between platelet mediated-hemostasis, viral infection, and immunosuppression. Furthermore, the authors perceptively suggested that the higher circulating platelet levels in mice compared to other species may explain why mice are not susceptible to AVHF and offered a simple alternative model to study the pathophysiology of AVHF [52]. This new experimental strategy, together with other recently described models of genetically modified mice, will help to clarify the issues regarding the pathogenesis of AVHF [53,54,55,56].
